# Feasibility and Accuracy of Menstrual Blood Testing for High-risk Human Papillomavirus Detection With Capture Sequencing

**DOI:** 10.1001/jamanetworkopen.2021.40644

**Published:** 2021-12-23

**Authors:** Jingjing Zhang, Xun Tian, Ye Chen, Sisi Huang, Zifeng Cui, Rui Tian, Zhen Zeng, Wenjia Liang, Qifen Gong, Ronghua Shang, Zheng Hu, Chen Cao

**Affiliations:** 1 Department of Obstetrics and Gynecology, First Affiliated Hospital, Sun Yat-sen University, Guangzhou, China; 2 Department of Obstetrics and Gynecology, Academician Expert Workstation, Central Hospital of Wuhan, Tongji Medical College, Huazhong University of Science and Technology, Wuhan, China; 3 Medical Examination Center, Central Hospital of Wuhan, Tongji Medical College, Huazhong University of Science and Technology, Wuhan, China; 4 Center for Translational Medicine, First Affiliated Hospital, Sun Yat-sen University, Guangzhou, China; 5 School of Medicine, Jianghan University, Wuhan, China; 6 Sun Yat-sen University Nanchang Research Institute, Nanchang, China

## Abstract

**Importance:**

High-risk human papillomavirus (hrHPV) persistent infection is the major etiology of cervical precancer and cancer. Noninvasive self-sampling HPV testing is a promising alternative cervical cancer screening for avoiding stigma and improving patient willingness to participate.

**Objective:**

To investigate the feasibility and accuracy of menstrual blood (MB) hrHPV capture sequencing in hrHPV detection.

**Design, Setting, and Participants:**

This cohort study collected 137 sanitary pads from 120 women who were premenopausal and had hrHPV as detected by cervical HPV GenoArray testing. Patients were recruited from September 1, 2020, to April 1, 2021, at Central Hospital of Wuhan, China. Target capture sequencing was performed to determine hrHPV genotypes in MB. Sanger sequencing was performed as the criterion standard for detecting hrHPV genotypes among enrolled women. Data were analyzed from April 1 through June 1, 2021.

**Main Outcomes and Measures:**

Complete concordance, incomplete concordance, and discordance of MB hrHPV capture sequencing and conventional HPV testing were defined according to genotype overlapping levels. Concordance of the 2 detection methods and comparative power of MB hrHPV capture sequencing during different menstrual cycle days (MCDs) were the main outcomes.

**Results:**

A total of 120 enrolled women with hrHPV (mean [SD; range] age, 33.9 [6.9; 20.0 -52.0] years) provided 137 sanitary pads. The overall concordance rate of MB hrHPV capture sequencing and cervical HPV testing was 92.7% (95% CI, 88.3%-97.1%), with a κ value of 0.763 (*P* < .001). Among 24 samples with incomplete concordance or discordant results, 11 samples with additional hrHPV genotypes (45.8%), 5 true-negative samples (20.8%), and the correct hrHPV genotypes of 2 samples (8.3%) were correctly identified by MB hrHPV capture sequencing. MB hrHPV detection of hrHPV was equivalent on different MCDs, with an MB hrHPV–positive rate of 27 of 28 patients (96.4%) for MCD 1, 52 of 57 patients (91.2%) for MCD 2, 27 of 28 patients for MCD 3, 4 of 4 patients (100%) for MCD 4, and 3 of 3 patients (100%) for MCD 5 (*P* = .76). The sensitivity of the MB hrHPV capture sequencing was 97.7% (95% CI, 95.0%-100%).

**Conclusions and Relevance:**

These findings suggest that MB hrHPV capture sequencing is a feasible and accurate self-collected approach for cervical cancer screening. This study found that this method is associated with superior performance in identification of HPV genotypes and true-negative events compared with cervical HPV testing.

## Key Points

**Question:**

Is menstrual blood high-risk human papillomavirus (hrHPV) capture sequencing a feasible and accurate approach for HPV detection?

**Findings:**

In this cohort study of 120 women with hrHPV, menstrual blood hrHPV capture sequencing had a high concordance rate with cervical HPV testing and offered advantages in the detection of additional hrHPV genotypes and true negative samples.

**Meaning:**

These findings suggest that menstrual blood hrHPV capture sequencing is a feasible and accurate self-collected approach for hrHPV detection.

## Teaser

This cohort study compares high-risk human papillomavirus (HPV) detection using menstrual blood testing with capture sequencing vs cervical HPV testing.

## Introduction

Persistent infection of high-risk human papillomavirus (hrHPV) is associated with various HPV-related precancers and cancers.^1^ In recent years, HPV testing has gradually become the primary method for cervical cancer screening.^2^ Although cervical cancer screening has been found to be helpful in decreasing the incidence and mortality rate of cervical cancer, various factors (including medical infrastructure, culture and mentality, and society) may influence women’s acceptance of clinician sampling.^3^ Among women with overdue screening, 29% were afraid of the stigma and 14% were had fear of pain in 2 studies.^4,5^ Self-sampling HPV testing is a proposed alternative cervical cancer screening for avoiding stigma and improving participation.^6^ However, to our knowledge, most existing self-sampling HPV studies were based on various sampling brushes inserted into the vagina, and patients may experience discomfort during sampling.^7,8,9,10,11^ Compared with these methods, menstrual blood (MB) collection is associated with less stigma and pain.

MB offers a snapshot of cervical HPV infection status. Given that it is a biological fluid with periodic expulsion, MB is easy to collect. This suggests that HPV testing based on sanitary pads may be a convenient and noninvasive approach. More importantly, next-generation sequencing (NGS) for HPV detection, emerging as a highly sensitive method for HPV genotyping, has not yet been applied to MB HPV testing, to our knowledge. This study was designed to investigate whether MB hrHPV capture sequencing may be a feasible and accurate approach to detecting hrHPV infection among women who are premenopausal.

## Methods

Ethics approval was obtained from the institutional review board of the Central Hospital of Wuhan. All patients agreed to participate and provided signed informed consent before enrollment. This study is reported following the Strengthening the Reporting of Observational Studies in Epidemiology (STROBE) reporting guideline.

### Study Population

From September 1, 2020, to April 1, 2021, we recruited women who were premenopausal and had tested positive for hrHPV in the Central Hospital of Wuhan, China. Cervical smears were collected from recruited patients by an experienced gynecologist (C.C.). The 21 HPV GenoArray Diagnostic Kit (Hybribio) was used to detect hrHPV infection in cervical smears. This kit has been used in many cervical screening studies, with approval and recommendation from the China National Medical Products Administration for HPV testing.^12,13,14^ Residual cervical smears after HPV testing were collected and preserved at 4 °C until further Sanger sequencing.

**Figure 1.**
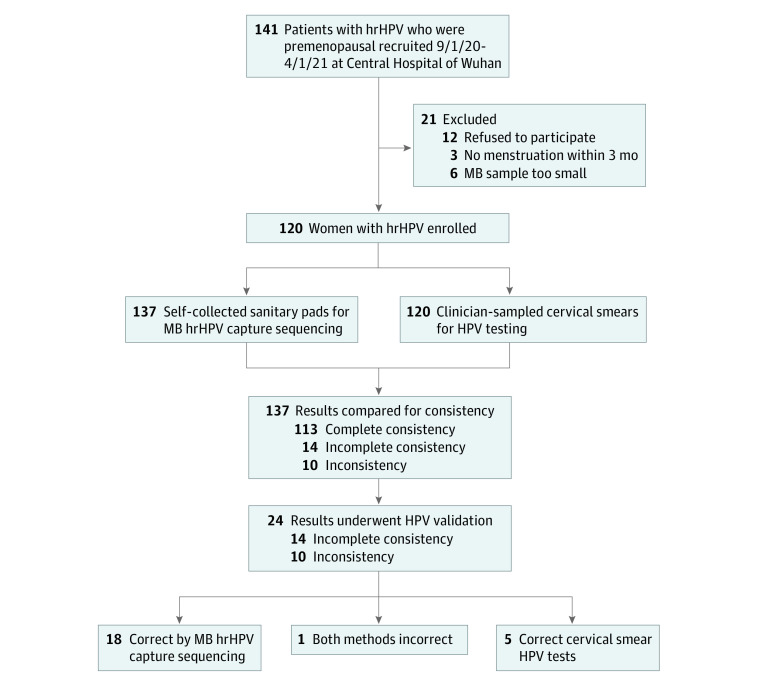
Study Flow Diagram HPV indicates human papillomavirus; hrHPV, high-risk HPV; MB, menstrual blood.

We excluded recruited patients who refused to participate in this study, had no menstruation within 3 months of hrHPV testing, or had a total menstrual bloodstain area on a sanitary pad of less than 3 cm × 3 cm (Figure 1). Enrolled patients were treated based on the 2019 American Society for Colposcopy and Cervical Pathology (ASCCP) consensus guidelines.^15^ The diagnosis of cervical intraepithelial neoplasia (CIN) and cervical cancer were confirmed by the pathology of cervical biopsies or excisions.

### Menstrual Sanitary Pads Collection

Self-provided commercial sanitary pads were used to collect MB from enrolled patients. The discarded sanitary pads were preserved in sterile sealed plastic bags that we distributed to enrolled patients in advance; pads were then placed in the refrigerator at −20 °C within 1 hour after being removed and stored until further testing. The menstrual cycle day (MCD) was recorded according to patient self-report. Patients were encouraged to provide multiple sanitary pads for different MCDs of their current menstrual cycle.

### MB DNA Extraction

A commercial Tiangen dried blood spot DNA extraction kit (Tiangen Biotech) was used to extract MB DNA from sanitary pads according to the manufacturer’s protocol. The concentration of DNA was confirmed using the Qubit 4.0 fluorometer and Qubit dsDNA high-sensitivity (HS) assay kit (Invitrogen by Thermo Fisher Scientific), and the purity of DNA was determined by NanoDrop One spectrophotometer (Thermo Fisher Scientific). Extracted MB DNA was stored at −20 °C.

### HPV Capture Hybridization

Whole-genome sequences of 15 types of hrHPV were obtained from GenBank. The accession numbers were as follows: NC_001526.4(HPV16), AY262282.1(HPV18), HQ537687.1(HPV31), HQ537688.1(HPV33), HQ537730.1(HPV35), LR862071.1(HPV39), EF202167.1(HPV45), LR862072.1(HPV51), HQ537751.1(HPV52), LR862079.1(HPV53), EF177181.1(HPV56), HQ537777.1(HPV58), EU918767.1(HPV59), EF177191.1(HPV66), and FR751039.1(HPV68). We designed a custom capture panel using the Integrated DNA Technologies (IDT) target capture probe design website and ordered the biotinylated RNA probe library from IDT. Probe sequences were subjected to deduplication, and those homologous to human sequences were removed. The resulting probe library comprised 120-nucleotide RNAs complementary to 1 strand of 1 of the 15 types of hrHPV at 2-fold coverage. Readers interested in the biotinylated RNA probe library should please contact the corresponding authors for more detailed information.

Mechanical disruption by Bioruptor Pico (Diagenode) was performed to shear MB DNA. Agencourt AMPure XP beads (Beckman Coulter) were used to purify sheared DNA fragments. MB DNA libraries were prepared using the TargetSeq enrichment kit (iGeneTech) according to the manufacturer’s protocol.

Hybridization capture with the previously listed 15 types of hrHPV probes was performed at 65 °C for 16 hours. After polymerase chain reaction (PCR) amplification, the concentration of PCR products was confirmed using Qubit 4.0 fluorometer and Qubit dsDNA HS assay kit (Invitrogen); the size of PCR products was confirmed using Qsep100 bioanalyzer (BIOptic). Products in the final library ranged from 270 base pairs to 320 base pairs in length. Sequencing was performed using Illumina NovaSeq PE150.

### HPV Genotyping and Validation

Our previous HPV detection reports^16,17^ provided details on the HPV genotyping method. Briefly, after filtering of low-quality reads and duplicated reads, all clean reads were aligned to the merged 15 hrHPVs whole-genome sequence. HPV-positive genotypes were then identified with mapping coverage of 50% or more and effective mean depth of ×30 or more. Next, we compared genotyping results of MB hrHPV capture sequencing with those of conventional HPV testing. Here, we defined the degree of detection concordance between MB hrHPV capture sequencing and conventional HPV testing as follows: if there was at least 1 overlapping hrHPV genotype between testing methods among all genotypes found in 1 patient's sample, it was defined as concordance. If the 2 were identical, it was defined as complete concordance; otherwise, it was defined as incomplete concordance. If there was no overlapping hrHPV genotype between testing methods, it was defined as discordance. Sanger sequencing of cervical smears was performed as the criterion standard for hrHPV genotypes (Figure 1).

### Statistical Analysis

Based on the results of previous studies, we estimated that MB HPV testing would yield 83% sensitivity and 98% specificity.^18^ Using a 10% allowable error, a 5% α error, and an estimated 90% prevalence of hrHPV infection among the targeted population, the minimum sample size was calculated to be 90 patients.

SPSS statistical software version 25.0 (IBM) was used for statistical analysis. Normal distribution data were shown as mean (SD). *P* value < .05 was considered statistically significant, and *P* values were 2-sided. Pearson χ^2^ test was used for comparison of the distributions of hrHPV genotypes detected by MB hrHPV capture sequencing and HPV testing. We also used Pearson χ^2^ test for comparison of MB hrHPV–positive rates on different MCDs. When sample sizes were too small to support Pearson χ^2^ test, Fisher exact test was used. We used κ test to analyze concordance of HPV detection methods. Sanger sequencing was used as the criterion standard for hrHPV genotypes.

## Results

### Patient Characteristics

A total of 120 patients with hrHPV detected by cervical HPV testing were enrolled (mean [SD; range] age, 33.9 [6.9; 20-52] years) among 141 recruited patients. Among all recruited patients, 12 individuals (8.5%) refused to participate in the study, 4 patients (2.8%) left Wuhan for job transfer, 3 patients (2.1%) were too busy to attend the study, 2 patients (1.4%) went to other hospitals for personal reasons, 2 patients (1.4%) lived too far away from our hospital to participate, and 1 patient (0.7%) did not provide a reason for not participating. We excluded 3 patients because they had no menstruation within 3 months of hrHPV testing and 6 patients because they had a menstrual bloodstain area of less than 3 cm × 3 cm.

By cervical HPV testing in the study population, 20 patients were positive for HPV 16 (16.7%), 12 patients were positive for HPV 18 (10.0%), and 93 patients were positive for other hrHPV genotypes (77.5%). The 3 most common hrHPV genotypes were HPV 52 (43 patients, [35.8%]), HPV 58 (26 patients, [21.7%]), and HPV 16. Multiple hrHPV infection was detected among 20 patients (16.7%).

There were 43 patients referred to colposcopy and cervical biopsies according to 2019 ASCCP consensus guidelines, and 5 patients underwent cervical conization. Additionally, CIN 1, CIN 2, and CIN 3 were confirmed in 7, 3, and 2 patients by pathology, respectively. There was no evidence of CIN or cervical cancer among the other 108 patients.

### Results of MB hrHPV Capture Sequencing

**Figure 2.**
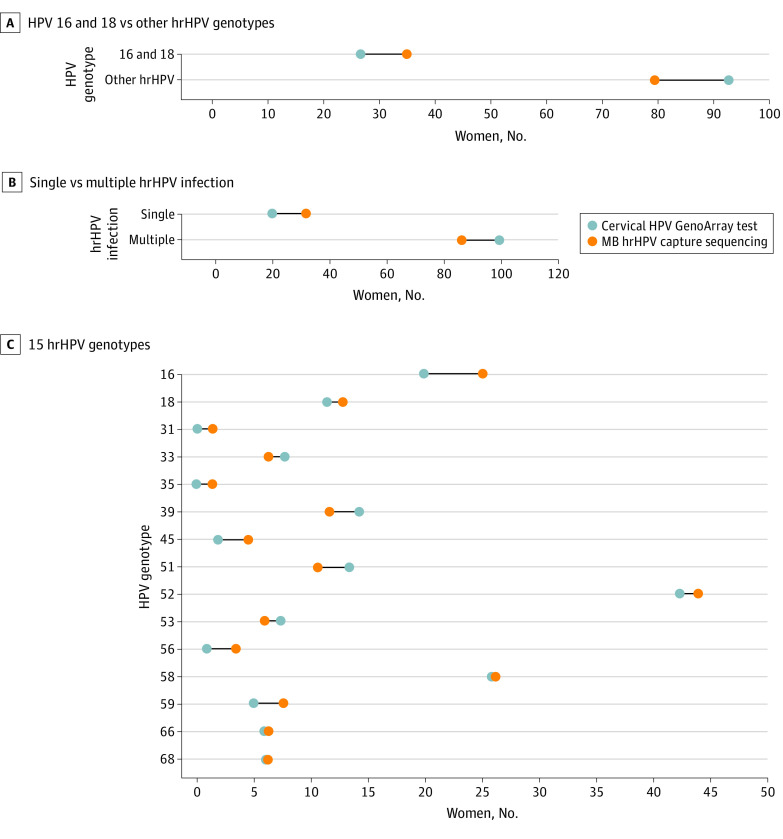
Distribution of Genotypes A, Human papillomavirus (HPV) 16 and HPV 18 are compared with other high-risk HPV (hrHPV) genotypes as detected by menstrual blood hrHPV capture sequencing and cervical HPV testing (*P* = .16); B, Single and multiple hrHPV infection as detected by menstrual blood hrHPV capture sequencing and cervical HPV testing are compared (*P* < .001); C, 15 hrHPV genotypes detected by menstrual blood hrHPV capture sequencing and cervical HPV testing are compared (*P* = .85).

There were 113 patients (94.2%) who tested positive for MB hrHPV by capture sequencing. In MB hrHPV capture sequencing, 22 patients were positive for HPV 16, 12 patients were positive for HPV 18, and 79 patients were positive for other hrHPV genotypes. Distributions of HPV 16 and HPV 18 vs other hrHPV genotypes were not significantly different in cervical HPV testing (27 patients vs 93 patients) compared with MB hrHPV capture sequencing (34 patients vs 79 patients) (*P* = .16) (Figure 2A). In MB hrHPV capture sequencing, the 3 most common hrHPV genotypes were HPV 52 (37 patients [32.7%]), HPV 58 (22 patients [19.5%]), and HPV 16 (22 patients [19.5%]). Multiple hrHPV infection was detected among 29 patients (25.7%) by MB hrHPV capture sequencing. The multiple hrHPV infection rate was statistically significantly different between detection methods (*P* < .001) (Figure 2B). Distributions of hrHPV genotypes between detection methods were not statistically significantly different (*P* = .85) (Figure 2C).

### MB hrHPV Capture Sequencing on Different MCDs

There were sanitary pads from 28 patients (23.3%) for MCD 1, 57 patients (47.5%) for MCD 2, 28 patients (23.3%) for MCD 3, 4 patients (3.3%) for MCD 4, and 3 patients (2.5%) for MCD 5. The sanitary pads of MCD 2 were preferred because there was typically a larger amount of bleeding.

The MB hrHPV–positive rate was 27 of 28 patients (96.4%) for MCD 1, 52 of 57 patients (91.2%) for MCD 2, 27 of 28 patients for MCD 3, 4 of 4 patients (100%) for MCD 4, and 3 of 3 patients (100%) for MCD 4. The MB hrHPV–positive rates for different MCDs were not statistically significantly different (*P* = .76).

**Figure 3.**
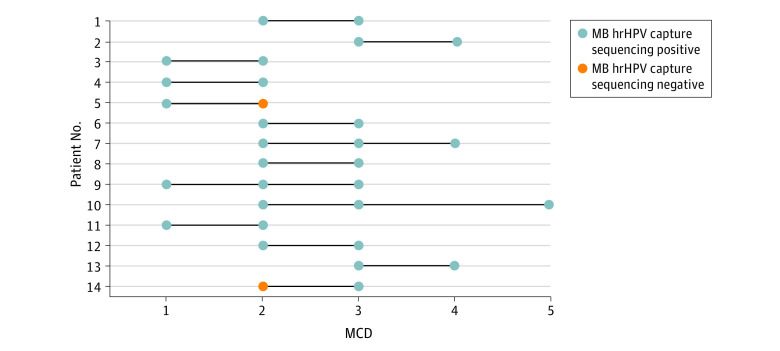
Testing by Menstrual Cycle Day (MCD) Sanitary pads of 14 patients were collected at multiple MCDs. There were 12 patients who tested positive for menstrual blood high-risk human papillomavirus (MB hrHPV) for each MCD on which they were tested, while patient 5 tested positive for MB hrHPV at MCD 1 but not MCD 2 and patient 14 tested positive for MB hrHPV at MCD 3 but not MCD 2.

Additionally, 14 patients provided 2 or 3 sanitary pads from different MCDs during a single menstrual period. Among these individuals, 12 patients (85.7%) tested positive by MB hrHPV for each MCD, and the overall MB hrHPV–positive rate for any MCD was 14 patients (100%) (Figure 3). There was no statistically significant difference in MB hrHPV–positive rate between patients providing sanitary pads from multiple MCDs and patients providing sanitary pads from 1 MCD (100 of 106 patients [94.3%]) (*P* = .71).

### Concordance Analysis

A total of 137 sanitary pads were provided by 120 enrolled patients. The complete concordance rate of the detection methods was 113 pads (82.5%), and the incomplete concordance rate was 14 pads (10.2%). Thus, the overall concordance rate was 92.7% (95% CI, 88.3%-97.1%), with a κ value of 0.763 (*P* < .001).

A total of 24 sanitary pads had incomplete concordance or discordance for the detection methods (Table 1). Among individuals who provided these pads, 5 patients (20.8%) were correctly detected by conventional HPV testing and 18 patients (75.0%) were correctly detected by MB hrHPV capture sequencing. In 1 of these patients (4.2%), neither detection method correctly detected the genotype.

**Table 1.** hrHPV Genotypes Validated by Sanger Sequencing

Correct method^a^	hrHPV genotype		
	MB hrHPV capture sequencing	Cervical HPV testing	Sanger sequencing
MB hrHPV capture sequencing (n = 18)	HPV 52, 56	HPV 52	HPV 52, 56
	HPV 16, 51, 59	HPV 51	HPV 16, 51, 59
	HPV 39, 52, 68	HPV 39, 52	HPV 39, 52, 68
	HPV 16, 52	HPV 52	HPV 16, 52
	HPV 16, 58	HPV 16	HPV 16, 58
	HPV 16, 52, 68	HPV 52, 68	HPV 16, 52, 68
	HPV 18, 33	HPV 33	HPV 18, 33
	HPV 18, 52, 58	HPV 18, 58	HPV 18, 52, 58
	HPV 35, 45, 51, 52, 53, 58	HPV 51	HPV 35, 45, 51, 52, 53, 58
	HPV 51, 52	HPV 51	HPV 51, 52
	HPV 16, 52	HPV 52	HPV 16, 52
	Not detected	HPV 39	No HPV 39
	Not detected	HPV 58	No HPV 58
	Not detected	HPV 53	No HPV 53
	Not detected	HPV 39	No HPV 39
	Not detected	HPV 33	No HPV 33
	HPV 58	HPV 51	HPV 58
	HPV 31	HPV 52, 68	HPV 31
Cervical HPV testing (n = 5)	HPV 16, 59	HPV 16	HPV 16
	HPV 18, 45	HPV 18	HPV 18
	HPV 51, 52, 56	HPV 51	HPV 51
	Not detected	HPV 52	HPV 52
	Not detected	HPV 58	HPV 58
Both incorrect (n = 1)	Not detected	HPV 51, 53, 58	HPV 51, 58

Abbreviations: HPV, human papillomavirus; hrHPV, high-risk HPV; MB, menstrual blood.

^a^ Each row indicates the results for a specific individual with these 3 testing methods.

According to results of Sanger sequencing, MB hrHPV capture sequencing successfully identified 11 samples with additional hrHPV genotypes (45.8%), 5 true-negative samples (20.8%), and the correct hrHPV genotype of 2 samples (8.3%). However, MB hrHPV capture sequencing also yielded 3 false-positive results and 3 false-negative results. Among 11 samples with additional hrHPV genotypes, conventional HPV testing missed 4 HPV 16 infections and 1 HPV 18 infection. Missed detection of HPV 16 or 18 may be associated with omission of colposcopy that should have been performed. Among 6 samples with false-positive or false-negative results detected by MB hrHPV capture sequencing, none were misidentified as HPV 16 or HPV 18 (Table 1).

Overall, MB hrHPV capture sequencing produced 126 true positive events, 3 false positive events, 3 false negative events, and 5 true negative events. The method had a sensitivity of 97.7% (95% CI, 95.0%-100%) (Table 2).

**Table 2.** Results of MB hrHPV Capture Sequencing vs Sanger Sequencing

MB hrHPV capture sequencing result	Sanger sequencing result	
	Positive	Negative
Positive	126	3
Negative	3	5

Abbreviations: hrHPV, high-risk HPV; MB, menstrual blood.

## Discussion

According to the ASCCP consensus guidelines, it is recommended that women aged 25 to 65 years undergo cervical cancer screening; this population largely overlaps with the population of women at childbearing age.^15^ Menstruation is a physiological bleeding process during childbearing age. Tampons, sanitary pads, and menstrual cups used by women during menstrual periods may be adapted as convenient medical devices to collect MB. In this cohort study, 8.5% of recruited patients refused the invitation to participate in the study without giving any reasons, suggesting that the approach using tampons, sanitary pads, and menstrual cups was associated with decreased practical and psychological barriers to undergoing cervical cancer screening. Even in India, where there are several negative myths associated with menstruation, a study^19^ reported that 54% to 76% of eligible women volunteered to provide their menstrual pads and participate in MB HPV testing.

Accurate hrHPV genotyping may play a crucial role in risk stratification and appropriate management of hrHPV. Compared with conventional HPV genotyping methods, NGS was associated with detection of HPV genotypes with high sensitivity.^20,21^ To our knowledge, our study was the first to detect MB HPV with NGS technology and found a high sensitivity of 97.7%. Moreover, strengths of capture sequencing included detection of additional hrHPV infections and multiple hrHPV infections, identification of true-negative samples, and identification of the real hrHPV genotypes among those misidentified by conventional HPV testing. These results were all validated by a third testing method, Sanger sequencing.

Our study also found that MB hrHPV–positive rates on different MCDs, from MCD 1 to MCD 5, were equivalent. Additionally, MB hrHPV–positive rates were similar among patients who provided 1 sanitary pad and those who provided more than 1 sanitary pad. These findings suggest that self-collected MB at any day of the menstrual cycle is acceptable, which also suggests the convenience of MB hrHPV capture sequencing. However, we recommend using 1 sanitary pad from MCD 2 for MB hrHPV capture sequencing because of this day usually has a larger amount of bleeding.

### Limitations

There are several limitations to this study. First, self-collection of MB could be performed only during the menstrual period; thus, self-sampling time was subject to certain restrictions. Second, there were slight differences in the materials used to make the sanitary pads. We found that certain materials may affect DNA extraction. Third, the proportion of women who did not have hrHPV and who had CIN or cervical cancer was relatively low in our study. Expanding the sample size and further exploring the value of MB hrHPV capture sequencing in the detection of women who do not have hrHPV and have CIN or cervical cancer is needed in future studies.

## Conclusions

This study’s findings suggest that sanitary pads with MB may serve as medical devices for HPV detection. With the obvious convenience of this method, MB hrHPV capture sequencing may be associated with decreased stigma of gynecological examination and increased participation in cervical cancer screening. MB hrHPV capture sequencing was associated with superior performance compared with cervical HPV testing not only in detection of additional hrHPV infections and multiple hrHPV infections, but also in identification of true negative events and hrHPV genotypes misidentified by conventional HPV testing. These results suggest that MB hrHPV capture sequencing may be a feasible and accurate self-collected approach for cervical cancer screening.
